# Successful removal of a giant intrathoracic lipoma: a case report and review of the literature

**DOI:** 10.1186/1757-1626-1-87

**Published:** 2008-08-12

**Authors:** Ramona M Hagmaier, Glenn A Nelson, Larkin J Daniels, Adam I Riker

**Affiliations:** 1 Mitchell Cancer Institute-University of South Alabama, 1 Mobile Infirmary Circle, 1st Floor, Mobile, Alabama, 36607-3522, USA; 2 Gulf Regional Pathologists, Mobile Infirmary Medical Center, Mobile, Alabama, 36607, USA; 3 Cardiothoracic and Vascular Surgical Associates, 1855 Springhill Avenue, Mobile, AL, 36607, USA

## Abstract

We report a case of a 44-year old female who presented to her physician complaining of mild dyspnea. A follow-up chest X-ray and chest computed tomography scan revealed a giant bilateral intrathoracic mass, filling the right thoracic cavity and extending across the anterior mediastinum into the left chest cavity. This large mass caused a marked shift in the midline structures, displacing the heart to the left hemi-thorax. The patient underwent surgical removal of the thoracic and breast mass, with histologic examination confirming the diagnosis of a giant intrathoracic lipoma, weighing 4,320 grams and measured 34 × 28 × 11 cm. It is the largest intrathoracic lipoma documented in the modern literature.

## Case presentation

**Figure 1:**
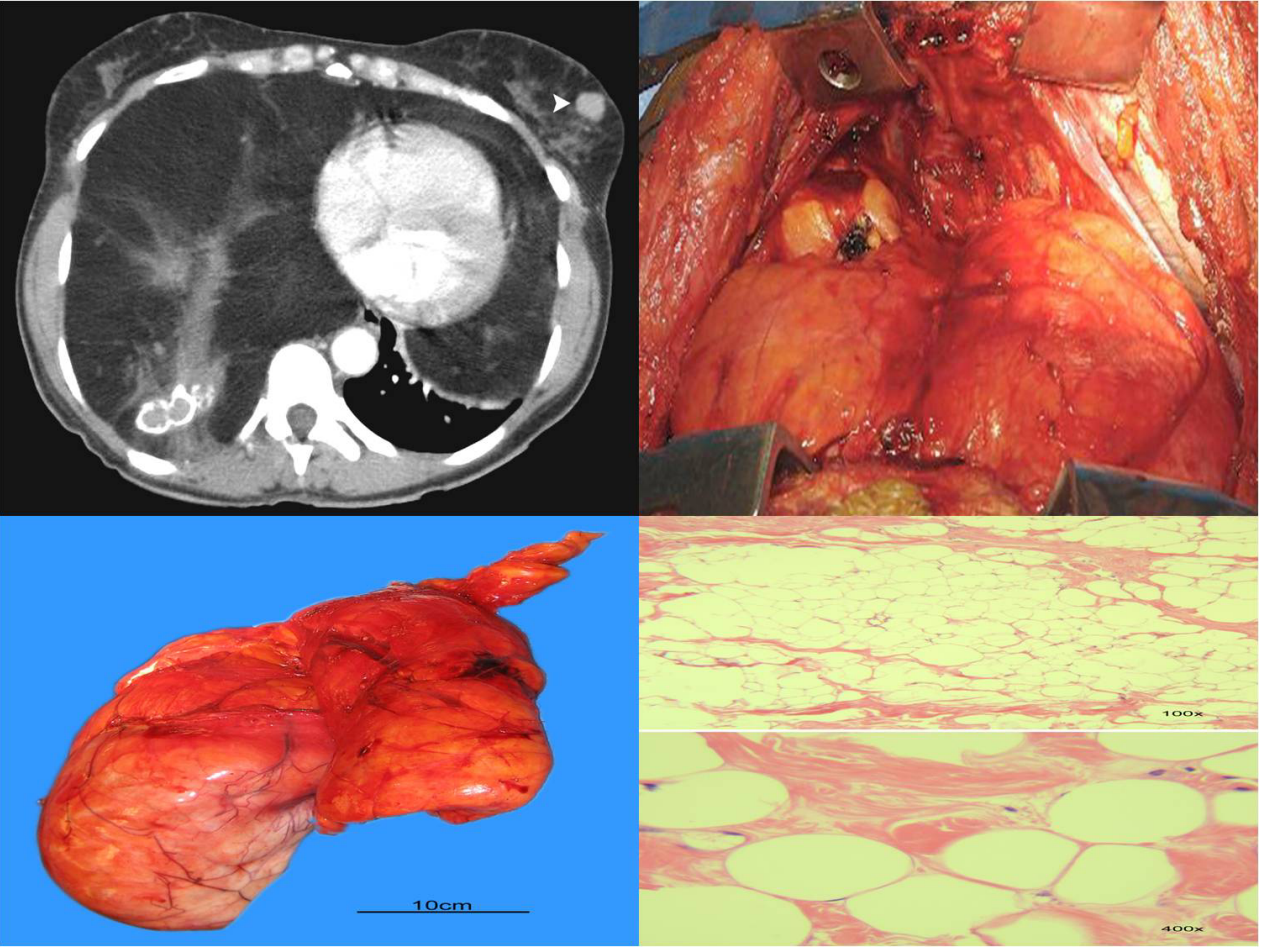
**A: Computed Tomography scan revealing large mediastinal mass**. **B: **Mediastinal mass extending into both thoracic cavities. **C: **Entire mediastinal mass after surgical extirpation. **D**: Histological features consistent with lipoma.

A 44-year old African American female who worked as a phlebotomist presented to her primary care physician complaining of mild shortness of breath and a 4-pillow orthopnea for many years (>10 years). She was a non-smoker, and maintained a healthy weight of 142 pounds for her 68" height. Her past medical history was significant only for uterine fibroids, for which she had undergone a myomectomy. She had conceived six healthy children, and the remainder of her family had no health problems. A subsequent chest x-ray and CT scan revealed a large mediastinal mass which occupied the majority of the right thoracic cavity and further extended across the anterior mediastinum into the left thoracic cavity (figure 1A). The posteroinferior portion of the mass revealed several areas of dystrophic ring-type calcifications within a field of scattered dense soft tissue elements. These were thought to possibly represent a pleural-based liposarcoma, lipoma, teratoma, teratocarcinoma, or invasive thymoma of the anterior mediastinum. The mass shifted the heart and mediastinal vascular structures to the left of midline.

In order to remove this large intrathoracic mass, the patient was explored through a transverse sternothoracotomy (clam-shell incision) to allow complete access to both pleural spaces and the mediastinum. Upon exposure of the anterior mediastinum, a large mass was visualized crossing this area and extending into both thoracic cavities (figure 1B). The mass further extended into the left thoracic cavity and displaced the lower lobe of the left lung. The gross appearance was that of a thinly encapsulated, dull mass with a pale yellow color which had a smooth surface and was soft to the touch. After a period of blunt dissection and manipulation, the entire mass was successfully removed en-bloc (figure 1C). The tumor weighed a total of 4,320 grams and measured 34 × 28 × 11 centimeters.

The patient's postoperative course was complicated by the development of several partially gangrenous and necrotic toes bilaterally, with the right toes affected greater than the left toes. A full hypercoagulation work-up was negative, and no evidence of arterial or venous thrombosis was ever identified. It is strongly suspected that this occurred as the result of a fatty embolic event related to the removal of the large tumor mass. This, in turn, required a partial amputation of the distal portion of 5 toes on the right and 2 toes on the left. Approximately 3 months after her initial hospitalization and surgery, the patient had complete resolution of her shortness of breath and was ambulatory.

Final histologic analysis of the tumor revealed an encapsulated tumor with abundant mature adipose tissue, a few scattered strands of fibroconnective tissue, multiple foci of calcification and fat necrosis. The bland histological features were consistent with the diagnosis of a lipoma (figure 1D). Liposarcoma was ruled out due to the absence of increased cellularity and lack of atypical nuclei. Thymolipoma was considered, however there was no evidence of thymic tissue within the specimen. The final diagnosis was that of a giant mediastinal lipoma.

## Discussion

Clinically, the finding of a lipoma is very common, mostly found within the subcutaneous areas of the body. These are almost always benign in nature with only rare malignant degeneration reported anecdotally. Such benign findings within the thoracic cavity are extremely uncommon, with only rare reported cases of giant intrathoracic masses reported. These well-circumscribed mesenchymal tumors originate from adipose tissue, are fully encapsulated in most cases, and are typically very slow growing, often over many years [1]. They are usually identified by routine chest radiograph or are discovered after the patient presents with symptoms of shortness of breath with exertion or dyspnea secondary to the compression of the bronchi, vagus nerve, esophagus, or other internal structures. Other symptoms can include cough, arrhythmias, orthopnea and intermittent dysphagia [2]. Jack et al. reports a case of an intrathoracic, extrapericardial lipoma which presented in a patient with severe left ventricular dysfunction [3]. The patient refused surgical extirpation of the tumor and subsequently suffered cardiac arrest due to the direct compression upon the heart from this large mass [3,4].

Current recommendations include complete en-bloc removal of such tumors whenever possible, as this is the only definitive treatment option. Once resected, local recurrence of intrathoracic or mediastinal lipomas is uncommon. Mediastinal lipomas typically arise within the anterior mediastinum and represent only 1.6–2.3% of all primary mediastinal tumors [1]. Histologically, lipomas are composed of sheets of mature adipocytes separated by fibrous, incomplete septa. Differentiating a lipoma from a liposarcoma can be challenging in many cases, especially if it is a low-grade malignancy. Benign lipomas consist of mostly mature adipose cells with no mitotic activity, while liposarcomas have adipose cells of varying size with hyperchromatic nuclei and eosinophilic cytoplasm. Liposarcomas can also have a varying number of mitoses associated with multinucleated histiocytes and fatty necrosis seen in 25% of cases [5]. Thymolipoma is another benign, slow growing neoplasm of the mediastinum. Microscopic examination may reveal thymic epithelium and/or multi-laminated keratinized cells arranged around clusters of degenerating lymphocytes (Hassall's corpuscles) [6].

## Consent

Written informed consent was obtained from the patient for publication of this case report and accompanying images. A copy of the written consent is available for review by the Editor-in-Chief of this journal.

## Competing interests

The authors declare that they have no competing interests.

## Authors' contributions

RH assisted with the surgery, wrote the case report, performed the literature review and obtained informed consent, AR and LD carried out the patient diagnosis, performed the surgery, removed the mass, and were major contributors in drafting the manuscript, AR and RH were involved in the patient's post operative management, GN conducted the histopathology and provided the photographs. All of the authors were involved in the patient's care and all authors read and have been involved in approving the final manuscript.

## References

[B1] Gaerte SC, Meyer CA, Winer-Muram HT, Tarver RD, Conces DJ (2002). Fat-containing lesions of the chest. Radiographics.

[B2] Cutilli T, Schietoma M, Marcelli VA, Ascani G, Corbacelli A (1999). Giant cervico-mediastinal lipoma. A clinical case. Minerva Stomatol.

[B3] Jack AI, Blohm ME, Lye M (1995). An intrathoracic lipoma impairing left ventricular function. Br Heart J.

[B4] Vougiouklakis T, Mitselou A, Agnantis NJ (2006). Giant Lipoma: An unusual cause of intrathoracic mass. Pathol Res Pract.

[B5] Weiss SW, Enzinger GoldblumJR (1994). Lipomatous Tumours. Histological Typing of Soft Tissue Tumours.

[B6] Romero-Guadarrama MB, Duran-Padilla MA, Cruz-Ortiz H, Castro-Gomez L, Lopez-Vancell D, Novelo-Retana V, Santiago-Prieto AC, Fierro-Chavez E, Rodríguez-Martinez HA (2004). Diagnosis of thymolipoma with fine needle aspiration biopsy. Acta Cytologica.

